# Use of Horse Chestnut (*Aesculus hippocastanum L*.) Starch in Gluten‐Free Cakes: Physicochemical, Nutritional, Textural Properties, and Determination of Pore Structure Using Conventional Thresholding Algorithms

**DOI:** 10.1111/1750-3841.70243

**Published:** 2025-05-07

**Authors:** Ali Cingöz

**Affiliations:** ^1^ Department of Food Engineering Tokat Gaziosmanpasa University Tokat Turkey

**Keywords:** alkaline/ultrasonic methods, celiac, gluten‐free, image processing, starch

## Abstract

**Abstract:**

Research into alternative starch sources for the production of gluten‐free products continues. In this study, starch production from horse chestnut seeds was carried out using alkali and ultrasound‐assisted methods, and the starches produced were used in the production of gluten‐free cakes. The obtained horse chestnut starches were used in the preparation of gluten‐free cakes and compared with gluten‐free cakes prepared with rice, maize, and potato starches. The physical quality parameters of the gluten‐free cakes were determined using image processing methods. The chemical, nutritional, and textural properties of the gluten‐free cakes were also determined. Physical, chemical, nutritional, and textural properties of gluten‐free cakes were determined. After 28 days of storage, the hardness values of gluten‐free cakes ranged from 50.13 to 68.41 N, and the springiness values ranged from 36.28% to 47.34%. The RDS values of horse chestnut starch and gluten‐free cakes were found to be 37.71% and 32.76%, respectively. The pore structures (cell count, total area, mean cell size, cell periphery, and fractal distribution) of gluten‐free cakes were determined using five different thresholding algorithms (Huang, MaxEntropy, Intermodes, Isodata, and Otsu). Gluten‐free cakes made with horse chestnut starch were similar to rice starch in terms of physical and textural properties, maize starch in terms of slowly digestible starch and PGI, and maize and rice starch in terms of pore structure. The Huang, Isodata, and Otsu algorithms were more effective in determining the pore structure of gluten‐free cakes. These results suggest that horse chestnut starch may be a promising alternative for use in gluten‐free products.

**Practical Application:**

Pore structure is one of the most important quality criteria in products such as cakes and bread. The pore structure determined by different methods is not efficient due to the disadvantages of the methods. The pore structure of cakes has been successfully determined by thresholding algorithms. Huang, Isodata, and Otsu algorithms showed more successful results. In addition, an alternative starch source for the production of gluten‐free products is proposed.

## 1 Introduction

*Aesculus hippocastanum* L. is a common ornamental plant in Turkey and many European and North American cities. Municipalities must dispose of tons of shredded and fallen fruit from this large tree. This disposal process needs to be done properly. This increases the cost considerably (Gullón et al. 2020). Therefore, researchers have focused on converting waste into value‐added products to reduce waste and environmental problems (Molina‐Moreno et al. 2017). In this context, there are studies on the production of high value‐added products such as antioxidants, glucose, and lignin from horse chestnuts (Gullón et al. 2020). *A. hippocastanum* L. (horse chestnut) has been widely used as an ornamental tree in parks, gardens, and boulevards since the end of the 16th century with the decorative properties of its leaves and flowers. It is also a valuable medicinal plant whose organs, such as seed pods and flowers, are used in traditional and medical treatments. The main bioactive constituents of horse chestnut, an important source of starch and oil, have been identified as 190 different constituents, including triterpene saponins (escin), flavonoids, coumarins, and proanthocyanidins (Amiri et al. 2019; Owczarek‐Januszkiewicz et al. 2023). The FDA classified hcrumorse chestnut seed as unsafe because it contains glycosides and saponins (FDA 2023). Today, horse chestnuts are widely cultivated in temperate regions as ornamental and landscape trees and are used as animal feed. There is evidence that Indians included it in their diet (Dudek‐Makuch and Matławska 2011). The fruit is used in the treatment of many diseases (Dridi et al. 2023). Anti‐nutritional factors are known to damage the biological system by interfering with the availability of nutrients in the body. Various pre‐treatments can eliminate or reduce anti‐nutritional factors in food crops (soaking, heat treatment, use of chemicals, etc.) (Kaur et al. 2012). Soaking and ultrasound‐assisted methods have eliminated or minimized toxic factors in horse chestnut seeds. The literature review includes studies on starch production from chestnut and horse chestnut using the alkaline method (Castaño et al. 2014; Correia and Beirão‐da‐Costa 2012; Lemos et al. 2015; Rafiq et al. 2016; Shah et al. 2016). There are studies investigating the effect of the ultrasonic‐assisted method on the properties of starch particles in starch production (Ahmad et al. 2020).

Studies focusing on quality improvement in products such as gluten‐free bread and cakes are very limited (Amani et al. 2022). The most important quality criteria in bakery products are color, shape, and crumb pore structure. Pore structure plays an important role in the evaluation of textural parameters (Rathnayake et al. 2018). Changes in pore structure affect the acceptability of gluten‐free cakes. Sensory evaluation, instrumental analysis, or the Dallman pore scales (TSE 1987) are used to evaluate the pore structure of cakes and breads. Due to the subjectivity of these methods and the formation of different pore structures with the use of alternative products, alternative methods are currently being investigated. Techniques and methods, such as hyperspectral imaging, ultrasound technology, and spectroscopy, have been investigated for this purpose, but the need for advanced equipment for these methods is a major issue (Grillo et al. 2014). Considering the diversity of pore structures in gluten‐free products and factors such as cost, alternative methods with high accuracy are needed. In this context, high‐resolution image scanners and cameras have been combined with computational analysis methods, and various algorithms and software have been developed to analyze pore structures (Tajima and Kato 2011). Thresholding is a simple but effective tool for extracting target features from the background gray values of pixels belonging to a particular object (Shi et al. 2023). Programs, such as Photoshop, ImageJ, and ArcGIS, are widely used for image segmentation based on the thresholding principle (Shi et al. 2023). Studies investigating the pore structure of bakery products are available in the literature (Jha et al. 2017; Ghaitaranpour et al. 2018; Rahimi et al. 2020).

Rice, potato, maize, and tapioca starches are the most commonly used in the production of gluten‐free cakes (Gómez 2022). Studies on the use of alternative starch sources such as green bean starch (Bassinello et al. 2020), sorghum starch (Curti et al. 2022), banana starch (Türker and Savlak 2022), *Pachyrhizus ahipa* root starch (Malgor et al. 2020), acha (*Digitaria exilis* stapf) starch (Deriu et al. 2022), and chestnut flour (Marciniak‐Lukasiak et al. 2022) in gluten‐free cake production are available in the literature. The aim of this study was to produce high value‐added starch from horse chestnut waste, which can be used as human food, to characterize the starches produced, and to investigate their potential for use in gluten‐free cake production. To this end, starch production and optimization were carried out using two different methods from horse chestnut, and the physicochemical, morphological, and thermogravimetric properties of the starch produced were determined. Horse chestnut starches were also used in the production of gluten‐free cakes and compared with rice, maize, and potato starches.

The literature review found no data on the use of horse chestnut starch in gluten‐free model food systems. Our study is new in terms of the use of horse chestnut starch in gluten‐free products and has the potential to fill this gap in the literature. In addition, it was noted from the literature that the studies carried out using imaging were on the pore structure of bread. The pore structure of gluten‐free cakes, which are softer and more prone to crumbling than bread, has not been assessed using these methods. The main research topic of this study is to evaluate, for the first time in the literature, the pore structure of gluten‐free cakes produced using horse chestnut starch and to determine the efficiency of the technique. Therefore, this research has the potential to make a significant contribution to the literature.

## 2 Materials and Methods

### 2.1 Materials

Horse chestnuts (*A. hippocastanum L*.) were used for starch extraction. Horse chestnut was obtained from parks and gardens in İstanbul (Turkey). The collected seeds were stored at −18°C until use.

### 2.2 Chemical Analysis

The moisture and ash content of the chestnut were determined according to AACC Standard Method Nos. 44‐01.01 and 08‐01.01, respectively (AACC 2004). Total starch was determined according to the AACC methods 76.13 using K‐TSTA kits of Megazyme (Megazyme Ireland Ltd., Co.). The micro‐Kjeldahl method was used to determine nitrogen contents (AOAC 2000). Crude fat was determined gravimetrically in an Ankom XT10 extraction system using the Filter Bag XT4 technique after petroleum ether extraction of the dried sample (Ankom Technology Inc., Macedon, NY) (AOCS 2005). The color of the samples was measured with a Minolta Chroma Meter (CR‐300 Minolta Japan).

### 2.3 Starch Extraction

Horse chestnut seeds were separated from the shell and cut into 3–4 mm thick pieces. The sliced pieces were soaked in 0.5% potassium metabisulphite and 1% citric acid solution for 30 min and dried at 70°C for 12 h (Singh et al. 2008). The parameters selected for the alkaline and ultrasonic extraction methods were determined through a literature review and preliminary experiments. For starch extraction by alkaline method, 100 g of sliced and dried horse chestnuts were added to 300 mL of 1% NaOH solution and kept at +4°C for 12 h. At the end of the time, the mixture was crushed in a high‐speed grinder for 5 min, passed through a 60 µm sieve, and the top of the sieve was washed 2–3 times with distilled water. The resulting filtrate was centrifuged (Boeco U‐32R, Germany) at 3000 rpm for 15 min. The centrifugation was repeated four times and washed with distilled water after each repetition. The supernatant obtained after the last centrifugation was collected in a large glass container, dried in an oven (Memmert100–800, Germany) at 40–50°C for 24 h, then ground and stored in sealed containers (Rafiq et al. 2015). For starch extraction by ultrasound‐assisted method, 100 g of horse chestnuts were added to 300 mL of 0.68% NaOH solution and treated in an ultrasonic water bath (Elma, E30H, Schmidbauer, Germany) for 2 h at 37 W ultrasound power. At the end of the time, starch production was realized by repeating the same procedures with the alkaline method.

### 2.4 Gluten‐Free Cake Production

The cakes were prepared by a modification of the AACC method 10–90 (AACC 2004) using starch (250 g), egg (100 g), sugar (180 g), milk (100 mL), sunflower oil (100 mL), vanillin (5 g), and baking agents (sodium pyrophosphate and sodium bicarbonate) (10 g). The starches used were rice (RS), maize (MS), potato (PS), horse chestnut starch produced by the alkaline method (A‐HCS), and horse chestnut starch produced by the ultrasound‐assisted method (U‐HCS). For the cake, eggs and granulated sugar were beaten for 5 min at speed 4 in a high‐speed mixer (Kitchenaid, USA). Milk and sunflower oil were added to the frothy mixture and beaten for a further minute at speed 3, then all the remaining solid ingredients were added and beaten for a total of 8 min. An amount of 150 ± 1 g of the prepared dough mixture was weighed and poured into pre‐greased special cake tins (10 × 13 × 5 cm). They were then baked in a convection oven (Kromlux KKFE/10, Turkey) at 180°C ± 2°C for 15 min. After removal from the oven, the cakes were taken out of the trays and left to cool at room temperature.

### 2.5 Physical Analysis

The cake's weights (g) and maximum heights (cm) were measured. AACC Standard Method No. 10‐05.01 (AACC 2004) was used to determine their volumes. The specific volume of the cake was calculated using the following Equation 1:

(1) $$\mathrm{Specific}\;\mathrm{volume}=\mathrm{volume}\mathrm{of}\mathrm{cake}\left(\mathrm{mL}\right)/\mathrm{weight}\mathrm{of}\mathrm{cake}\left(g\right)$$

The percentage of weight loss was calculated by measuring the weight of the cake before and after baking. The weight loss of the cake was calculated using the following Equation 2:

(2) $$\mathrm{Weight}\;\mathrm{loss}\;\left(\%\right)=\frac{\mathrm{Initial}\;\mathrm{weight}\;\mathrm{of}\;\mathrm{the}\;\mathrm{sample}-\mathrm{Final}\;\mathrm{weight}\;\mathrm{of}\;\mathrm{the}\;\mathrm{sample}\;\mathrm{after}\;\mathrm{baking}\;}{\mathrm{Initial}\;\mathrm{weight}\;\mathrm{of}\;\mathrm{the}\;\mathrm{sample}}\;\times 100$$

### 2.6 Color Measurement

The color of the crumb and crust of the cake samples was measured with a Minolta CR300 (Minolta Inc., Tokyo, Japan) using the Hunter L*, a*, and b* color scales. The Δ*E* value was calculated according to Equation (3), and the browning index (BI) (Srivastava et al. 2018) of the gluten‐free cake was calculated using the following Equation 4: In calculating the total colour change (ΔE) values, the L*, a* and b* values obtained from cakes made with rice starch were taken as reference values.

(3) $$\Delta E={\left[{\left(L-L_{\mathrm{ref}}\right)}^{2}+{\left(a-a_{\mathrm{ref}}\right)}^{2}+{\left(b-b_{\mathrm{ref}}\right)}^{2}\right]}^{1/2}$$

(4) $$\mathrm{BI}=\frac{\left[100\;\times \;\left(\frac{a^{*}+1.79L^{*}}{5.645L^{*}+a^{*}-3.012b^{*}}-0.31\right)\;\right]}{0.17}\;$$

### 2.7 Texture Profile Analysis

Cake samples were stored for 28 days, and texture profiles were analyzed on days 0, 7, 14, 21, and 28. Cake samples were packaged with a heat‐sealable three‐layer metallized film (20 µm thickness, oxygen transmission rate (23°C, 0% RH) (OTR) ≤150 cm^3^/m^2^/24 h, water vapor transmission rate (38°C, 90% RH) (WVTR) ≤1 g/m^2^/24 h, optical density 2.3% (Polimet 7514)) according to EC and FDA food contact standards 2 h after oven release. Packaged samples were stored in an oven at 25°C (Memmert 100‐800, Germany) for 28 days. For texture profile analysis, the method used by Gutiérrez‐Luna et al. (2024) was modified. The textural characteristics of the gluten‐free cakes, based on firmness and springiness, were measured using a TA‐XT2i texture analyzer (Texture Technologies Corp., Godalming, Surrey, UK) and Texture expert software (Version 2.03). Cake samples were analyzed whole and at room temperature. A P36/R cylindrical probe was used for measurement, and analysis was performed using a 50 kg load cell. In the analysis, the pre‐test speed was set to 1 mm/s, the test speed to 1 mm/s, the post‐test speed to 10 mm/s, the strain rate to 25%, and the tensile force to 5 g.

### 2.8 In Vitro Starch Digestibility

Nutritionally important starch fractions of cakes produced using five different starches were determined by in vitro digestion method (Englyst et al. 1992). Ground samples, 1.00 ± 0.01 g, were stirred with hydrolytic enzymes (pancreatin sigma P7545, amyloglucosidase, and invertase) at 160 rpm in a horizontal position in a water bath at 37°C. After 20 (G_20_) and 120 (G_120_) min, the tubes were removed, and the G_20_ and G_120_ fractions were centrifuged at 4000 × *g* for 10 min (Boeco U‐32R, Germany), and the glucose levels in the supernatants were determined by the glucose oxidase–peroxidase (GOPOD) method. The significant starch fractions were calculated using the following formulas.

$$\mathrm{TS}=\left(\mathrm{TG}-\mathrm{FG}\right)\;\times 0.9$$

$$\mathrm{RAG}=G_{20}$$

$$\mathrm{RDS}=(G_{20}\;-\mathrm{FG})\;\times 0.9$$

$$\mathrm{SDS}=(G_{120}\;-G_{20})\;\times 0.9$$

$$\mathrm{SHI}=\left(\frac{\mathrm{RDS}}{\mathrm{TS}}\right)\times 100$$

Total starch (TS), total glucose (TG), free glucose (FG), rapidly digestible starch (RDS), rapidly available glucose (RAG), slowly digestible starch (SDS), and starch hydrolysis index (SHI)

### 2.9 Predicted Glycemic Index (pGI)

The % total starch digested every thirty minutes between 0‐180 minutes was determined by the GOPOD method. The hydrolysis index (HI) value was determined from these values by a precise calculation method. The pGI of the starch was calculated according to the following equation 5 (Di Cairano et al. 2020):

(5) pGI=39.71+0.549×HI

### 2.10 Image Processing Method

**FIGURE 1:**
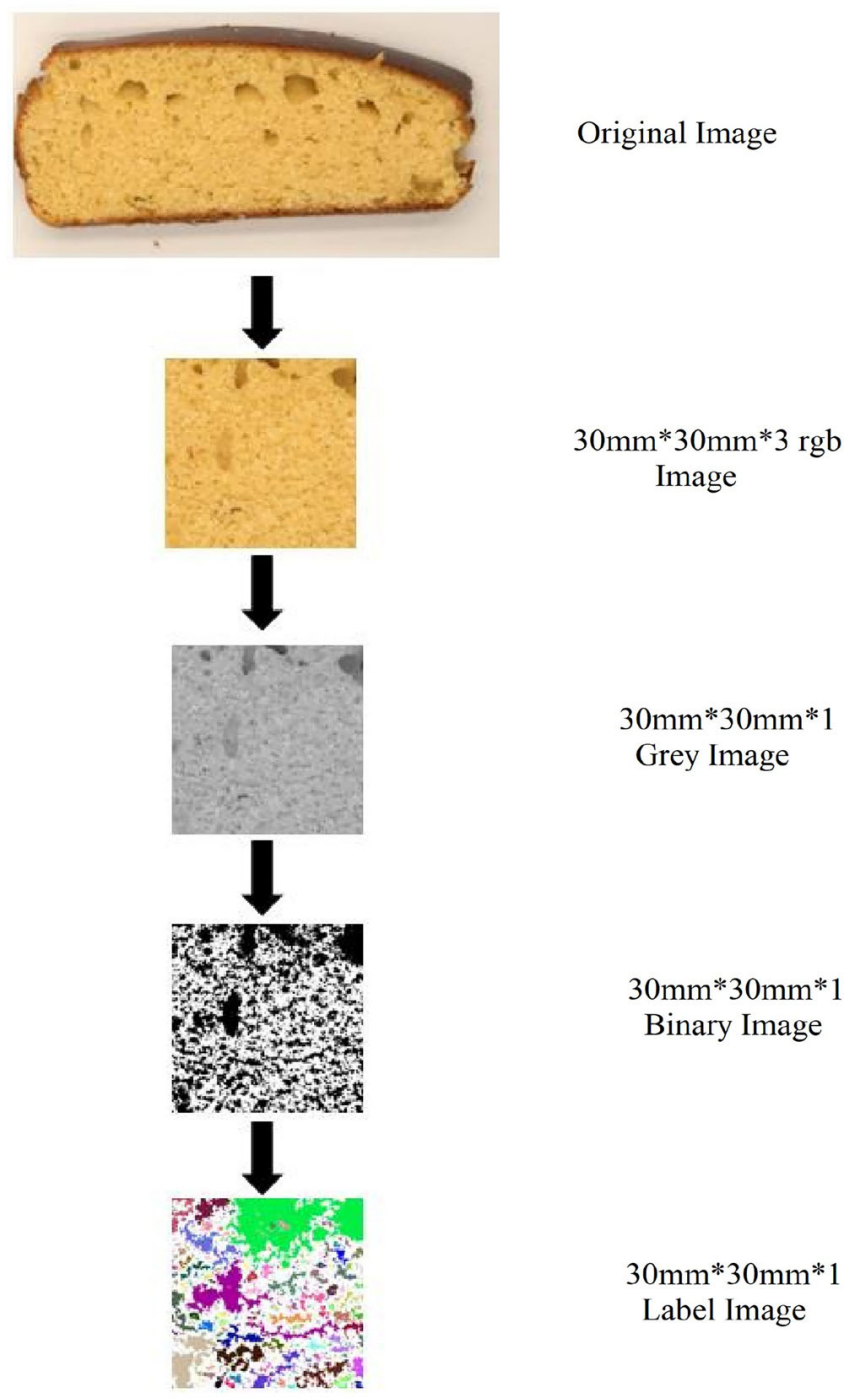
Image processing stages.

After cooling for 2 h at room temperature, the cakes were cut into equal pieces of 12–15 mm thickness vertically and horizontally, and two pieces were taken from the center. Photographs of the cake slices were taken in a professional mini‐shooting studio (5500 LM LED light sources, 100% power) with a high‐resolution camera (80D, DSLR, Canon) at a distance of 30 cm to scale and recorded lossless in RAW (6000 × 4000) format. Raw cake images were colored, and 16 (slice) × 2 (parallel) × 2 (repeat) = 64 cake images were obtained from each cake sample. The 64 × 5 = 320 cake images were converted to 300 dpi JPEG format using Adobe Illustrator software, and a 30 mm × 30 mm square colored field of view was cropped from each cake sample. The cut post‐color images were pre‐processed using ImageJ (Version 1.53) software and converted to 8‐bit gray scale. Each grayscale image file was processed using five different thresholding algorithms (Huang (Huang and Wang 1995), MaxEntropy (Kapur et al. 1985), Intermodes (Prewitt and Mendelsohn 1966), IsoData (Ridler and Calvard 1978), and Otsu (Otsu 1975)) and the pore structure of the cake was converted into 0–1 binary images. After removing the noise in the binary images, particle analysis was performed. The number of cells (objects), average cell area (mm^2^), and maximum and minimum cell area (mm^2^) were determined by particle analysis. The steps of the image processing method are shown in Figure 1. The general thresholding functions of the algorithms used are given in the following Equations (6 = Huang, 7 = Isodata, 8 = Otsu, 9 = Maxentropy, 10 = Intermodes) (Günen and Atasever 2024).

Huang general thresholding function:

(6) $$\begin{array}{ccc} T & = & \mathrm{argmin}\;\left(-\frac{1}{N^{2}\log2}\;\sum_{g=0}^{G}\left[\mu_{f\;}\left(g,T\right)\log\;\left(\mu_{f}\;\left(g,T\right)\right)\right]\right.\\ & & +\;\left.\left[1-\mu_{f\;}\left(g,T\right)\log\right]\;\log\;\left[1-\mu_{f\;}\left(g,T\right)\right]p\left(g\right)\right) \end{array}$$

Isodata general thresholding function:

(7) $$T=\lim_{n\to \infty }\frac{\mu_{f}\left(T_{n}\right)+\mu_{b}\left(T_{n}\right)\;}{2}\;$$

Otsu general thresholding function:

(8) $$T_{\mathrm{opt}}=\mathrm{argmax}\;\left(\frac{P\left(T\right){\left[1\;-\;P\left(T\right)]\;[m_{f}\left(T\right)\;-\;b\left(T\right)\right]}^{\left(2\right)}}{P\left(T\right)\sigma_{f}^{2}\left(T\right)+[1\;-\;P\left(T\right)\sigma_{b}^{2}\left(T\right)}\right)$$

Maxentropy general thresholding function:

(9) $$\;T_{\mathrm{opt}}=\mathrm{argmax}\;\left(-\sum_{g=0}^{T}\frac{\frac{p\left(g\right)}{p\left(T\right)}\log(p\left(g\right))}{p\left(T\right)}+\left(-\sum_{g=T+1}^{G}\frac{\frac{p\left(g\right)}{p\left(T\right)}\log(p\left(g\right))}{p\left(T\right)}\right)\right)$$

Intermodes general thresholding function:

(10) $$T=\frac{j+k}{2}$$

The fractal dimension of each cell (DFp) was calculated on the basis of the logarithmic relationship between the perimeter (*P*) (mm) and area (*A*) (mm^2^) of the cells using the following Equation 11 (Kenkel and Walker 1996; Olsen et al. 1993):

(11) DFp=2log(P/4)/log(A)

Similarly, the image entropy and texture fractal dimension proposed by Chen et al. (2003) are evaluated using Equation 12:

(12) $$\mathrm{DFt}=\log\left(N\right)\;\log\left(1/r\right)$$

*N* is the number of cells counted in different sizes and *r* is the size of the cell (mm^2^).

### 2.11 Statistical Analysis

The SPSS statistical program (SPSS Inc., Chicago, IL, USA) was used, analysis of variance of the results (ANOVA) was performed, and the differences between groups were statistically evaluated with a 95% confidence interval by the Duncan multiple comparison test.

## 3 Results and Discussion

The physical and chemical properties of horse chestnuts collected from park and garden wastes and used for starch production were determined. Seeds with 16.01 ± 2.09 g average seed weight and 2.44 ± 0.25 cm thickness had 78.78% ± 0.16%, 1.92% ± 0.03%, 4.85% ± 0.17%, 2.06% ± 0.17%, and 30.21% ± 0.46% dry matter, ash, protein, fat, and total starch contents, respectively. It has been reported that horse chestnut seeds contain 0.39%–7.78% protein, 1.12%–3.27% fat, 1.93%–3.16% ash, and 35.0%–38.3% starch (Cukanovi´c et al. 2011; Hassan et al. 2021; Rafiq et al. 2015, 2021; Shah et al. 2016). The composition of the horse chestnuts used is similar to that of the literature. Starch content was found to be lower than the literature. Factors, such as the collection time of horse chestnuts, climatic conditions, and variety, caused this difference.

### 3.1 Gluten‐Free Cakes

Table 1 shows the physicochemical characteristics, crust, and crumb structure of gluten‐free cakes made with five different starches. There is no significant difference between the baked weights of the samples (*p* < 0.05). It was found that the volume values ranged between 284.5 and 340.5 cm^3^/g, and the volume values of cakes produced using horse chestnut starch were similar to the volume values of cakes produced using rice starch (RS). This situation is also similar for the specific volume values. The swelling of the cake is caused by the air trapped in the cake batter during the beating of the eggs and sugar and is determined by the specific volume index (Majzoobi et al. 2014; Zhou et al. 2011). The cakes produced using maize starch (MS) had the highest cake height, and the heights of the cakes produced using A‐HCS and U‐HCS were 5.15 and 5.20 cm, respectively. The moisture content, which has a significant effect on the storage time and texture of the cakes, ranged from 23.25% to 25.56%, and there was no statistical difference (*p* < 0.05). The protein and fat contents of the samples are statistically similar (*p* < 0.05). The protein and fat contents of the samples varied between 2.33% and 2.45% and 16.04% and 16.25%, respectively. The crust thickness of the cakes varied between 2.58 and 2.75 mm. The crust thickness of the cakes produced with A‐HCS and U‐HCS was similar to the crust thickness of the cakes produced with MS and RS.

**TABLE 1:** Physicochemical characteristics of different gluten‐free cakes, their crumb, and crust properties.

Determination	Maize starch	Rice starch	Potato starch	Horse chestnut starch (alkaline)	Horse chestnut starch (ultrasound‐assisted)
Weight (g)	134.0 ± 1.9^b^	136.0 ± 1.3^a^	131.2 ± 2.7^bc^	132.7 ± 1.2^bc^	133.5 ± 2.7^b^
Volume (cm^3^)	340.5 ± 26.2^a^	309.6 ± 16^bc^	284.5 ± 14.1^c^	317.8 ± 15.9^b^	321.6 ± 14.1^b^
Specific volume (cm^3^/g)	2.55	2.29	2.18	2.40	2.42
Weight loss (%)	11	9.7	12.5	11.8	11.5
Height (cm)	5.35 ± 0.2^a^	5.0 ± 0.2^bc^	4.8 ± 0.1^c^	5.15 ± 0.1^b^	5.20±0.1^b^
Crust thickness (mm)	2.75 ± 0.12^a^	2.70 ± 0.30^a^	2.58 ± 0.08^b^	2.66 ± 0.08^a^	2.70 ± 0.14^a^
Moisture content (%)	24.93 ± 0.46^a^	25.56 ± 0.32^a^	23.25 ± 0.24^b^	25.12 ± 0.38^a^	25.23 ± 0.19^a^
Fat (%)	16.25 ± 0.23^a^	16.12 ± 0.33^a^	16.16 ± 0.18^a^	16.18 ± 0.21^a^	16.04 ± 0.28^a^
Protein (%)	2.45 ± 0.08^a^	2.33 ± 0.16^a^	2.36 ± 0.22^a^	2.44 ± 0.14^a^	2.42 ± 0.18^a^
Crumb characteristics	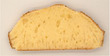	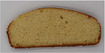	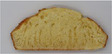	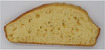	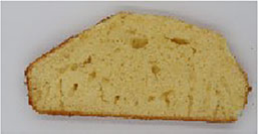
L*	74.5 ± 0.9^a^	68.8 ± 0.8^d^	70.0 ± 0.4^c^	71.1 ± 1.0^b^	70.7 ± 0.8^bc^
a*	−3.9 ± 0.1^b^	−3.7 ± 0.1^c^	−4.0 ± 0.1^a^	−3.5 ± 0.1^d^	−3.5±0.1^d^
b*	22.9 ± 0.4^d^	24.8 ± 0.6^a^	21.6 ± 0.7^e^	23.6 ± 0.5^c^	24.1 ± 0.4^b^
Δ*E*	6.01 ± 0.2^a^	‐	3.43 ± 0.1^b^	2.60 ± 0.1^c^	2.03±0.1^d^
Crust characteristics	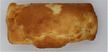	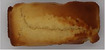	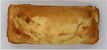	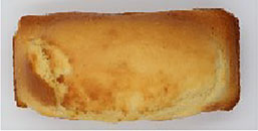	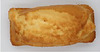
L*	55.7 ± 1.0^d^	56.7 ± 0.6^d^	66.2 ± 1.1^a^	61.2 ± 0.8^b^	60.3 ± 0.4^c^
a*	13.0 ± 0.8^a^	13.5 ± 0.4^a^	9.7 ± 0.6^b^	13.9 ± 0.7^a^	13.7 ± 0.6^a^
b*	38.6 ± 1.6^c^	40.7 ± 0.8^b^	45.9 ± 0.4^a^	38.1 ± 0.9^c^	37.8 ± 0.7^c^
Δ*E*	2.38 ± 0.2^d^	^‐^	11.48 ± 0.5^a^	5.21 ± 0.3^b^	4.63 ± 0.4^c^
BI	130.5 ± 2.6^c^	138.1 ± 1.8^a^	124.86 ± 1.6^d^	134.2 ± 1.9^b^	132.1 ± 1.4^c^

*Note*: The letters a, b, c, … indicate statistical differences at the *p* < 0.05 level of the samples in the same line; the values are means ± standard deviation of three replicates.

Abbreviation: BI, browning index.

The crumb and crust color characteristics of gluten‐free cakes were analyzed. The highest L* value was observed in cakes with MS, followed by cakes with A‐HCS, U‐HCS, and PS. The L* value of horse chestnut starch cakes was similar to that of PS cakes. The lowest a* value in the crumb color of the cakes was found in cakes with A‐HCS and U‐HCS. The crumb b* values of all cake samples are statistically different. The crumb and crust structures of the cakes and the differences between them are shown visually in Table 1. When the total color change (Δ*E*) values calculated for the crumb and crust of the gluten‐free cakes were examined, it was found that there was no difference between the cakes with A‐HCS and U‐HCS, and the highest total crumb and crust color change was found in the cakes with PS. Cakes with PS had the darkest crust color, followed by cakes with A‐HCS and U‐HCS. The crust a* values of all cakes except the PS cakes were found to be statistically similar (*p* < 0.05). For crust b*, cakes with A‐HCS and U‐HCS were statistically similar to cakes with MS (*p* < 0.05). Crust a* and b* values ranged from 9.7 to 13.9 and 38.1 to 45.9, respectively. The development of brown color is a result of Maillard and caramelization reactions, and the Maillard reaction is particularly emphasized. In some foods, such as bread, cakes, biscuits, and pizza, browning reactions occur at temperatures above 177°C (Soleimanifard and Akhavan 2024). The cake with the highest BI was the RS cake, followed by the A‐HCS and U‐HCS cakes. As the moisture content of the cake samples increased, the browning indices increased. As the moisture content decreases, molecular diffusion decreases, and consequently, the browning reaction and the rate of color change decrease (Rische and Leake 2020). The BI of cupcakes produced by adding protein isolate was reported to be in the range of 116.07–138.84 (Turker et al. 2024).

**FIGURE 2:**
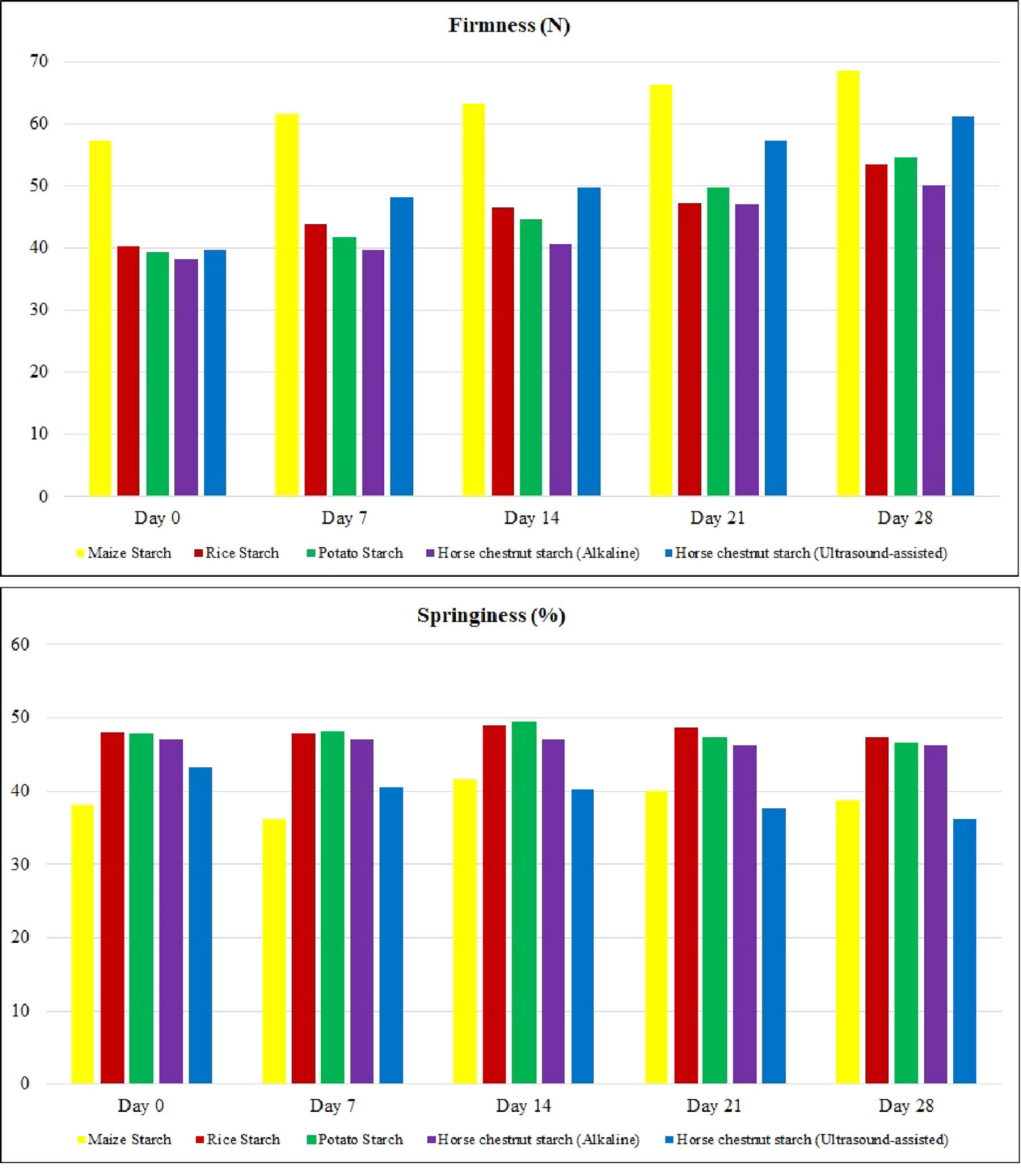
Texture values of gluten‐free cakes, 28 days.

Texture measurements during cake production and storage are important parameters used to quantify cake quality and to gain insight into cake staling (Lassoued et al. 2008). The 0–28 days firmness and springiness values of gluten‐free cake samples are shown in Figure 2. Firmness is the value of force required to achieve a given deformation and is given by the height of the first peak (Silva et al. 2020). The initial (Day 0) hardness values of the gluten‐free cakes varied between 38.12 and 57.11 N. MS cakes had the highest hardness value, whereas A‐HCS and U‐HCS cakes had similar hardness values to PS cakes. After 28 days storage, the hardness values of MS, RS, PS, A‐HCS, and U‐HCS cakes were 68.41, 53.36, 54.60, 50.13, and 61.22 N, respectively. Hardness is defined as the force required to compress the food with the molars during the first bite (Silva et al. 2020). The hardness increase rates of cakes with MS, RS, PS, A‐HCS, and U‐HCS were calculated to be 0.40, 0.47, 0.54, 0.43, and 0.77 N/day, respectively. It was found that the use of U‐HCS caused a faster increase in the hardness value. Prolonged ultrasonication of starch granules leads to a severe disruption of the crystalline structure of aggregated amylopectin and the formation of nanoparticles with low crystallinity or amorphous character (Haaj et al. 2013). This change in the starch granules is predicted to result in a faster increase in hardness value. When analyzing the texture values of cereal products such as cakes and bread, the springiness value is determined in addition to the firmness value. The springiness value is related to the elastic recovery between the first and second compression. The chewability values of the samples are calculated from the hardness and springiness values (Silva et al. 2020). The springiness values (%) of the cake samples are shown in Figure 2. The springiness values of all cake samples increased until day 14 and then started to decrease. The main reason for this situation is the crumbling problem that occurred in all samples. Cakes with RS, PS, and A‐HCS are statistically similar in day 0 springiness values (*p* < 0.05). At the end of day 28, the springiness values of MS, RS, PS, A‐HCS, and U‐HCS cakes were 38.82%, 47.34%, 46.54%, 46.22%, and 36.28%, respectively. Considering the texture values, it can be seen that A‐HCS is similar to RS and PS.

Starch, which is one of the basic raw materials in the production of gluten‐free products, is classified into fast and slow digestible starches according to the in vitro digestion rate and speed (Dona et al. 2010). Five nutritionally important starch fractions of gluten‐free cakes made with different starches were determined in vitro, and the results are shown in Table 2. Consumption of foods containing high levels of rapidly available glucose (RAG) disrupts the sugar regulation of metabolism by causing a sudden increase in blood glucose levels (Kylökäs et al. 2016). The RAG content of gluten‐free cakes was found to vary between 38.12% and 46.98%. The highest RAG content was found in cakes with RS, whereas the lowest was found in cakes with PS. Type 1 diabetes is a disease that is particularly common in celiac disease. The main reason for this is the need of people with celiac disease to consume a starch‐ and carbohydrate‐based diet (Kylökäs et al. 2016). For the prevention and control of metabolic diseases, especially Type 1 diabetes, foods with high SDS and low RDS should be preferred (Venn and Mann 2004). The lowest RDS content in gluten‐free cake samples was found in cakes with U‐HCS and PS. Potato starch contains more resistant starch and less RDS than other starches (Bodjrenou et al. 2023). The RDS content of A‐HCS and U‐HCS cakes was found to be 37.71% and 32.76%, respectively. Cakes produced with HCS are characterized by low RDS content. The SDS content of the cake samples ranged from 7.12% to 8.80%, and the highest SDS content was found in cake samples with MS and A‐HCS. The ratio of rapidly digestible starch to total starch in starchy foods is defined as the starch hydrolysis index (SHI). It relatively reflects the in vitro digestion rate and rate of starch in foods as well as the in vivo GI value (Englyst et al. 2003). SHI values of gluten‐free cakes ranged from 80.16 to 83.14. Gluten‐free cakes produced with horse chestnut starch by both methods had a lower hydrolysis index than other starches except potato starch. The positive effect of adding legume flours to a gluten‐free product formulation on reducing starch digestibility has been reported by many authors (Carboni et al. 2024; Gularte et al. 2012; Trevisan et al. 2019). However, no study was found in the literature that investigated the effect of alternative starch sources. Equations have been developed to estimate the GI based on in vitro starch hydrolysis (Goñi et al. 1997). The starch composition of products affects the estimated glycaemic index (pGI). The estimated GI was positively correlated with the amount of damaged starch, whereas flavonoids, amylose, and resistant starch (RS) content were negatively correlated with the pGI. The pGI values of the cake samples ranged from 78.48 to 80.46 (Table 2). The lowest pGI values were found in cakes with PS and A‐HCS. Rice flour has the highest pGI among all cereal flours (Di Cairano et al. 2020).

**TABLE 2:** Important starch fractions and predicted glycemic index (pGI) value of gluten‐free cakes.

Determination	Maize starch	Rice starch	Potato starch	Horse chestnut starch (alkaline)	Horse chestnut starch (ultrasound‐assisted)
**RAG (%)**	41.89 ± 0.12^c^	46.98 ± 0.23^a^	38.12 ± 0.21^e^	42.13 ± 0.18^b^	40.24 ± 0.16^d^
**RDS (%)**	37.12 ± 0.08^b^	41.57 ± 0.14^a^	35.23 ± 0.16^c^	37.71 ± 0.36^b^	32.76 ± 0.14^d^
**SDS (%)**	8.80 ± 0.08^a^	7.85 ± 0.06^c^	7.12 ± 0.10^d^	8.65 ± 0.22^a^	8.12 ± 0.21^b^
**SHI**	82.13	83.14	80.16	82.31	81.49
**pGI**	79.88	80.46	78.48	79.41	78.95

*Note*: The letters a, b, c, … indicate statistical differences at the *p* < 0.05 level of the samples in the same line; the values are means ± standard deviation of three replicates.

Abbreviations: pGI, predictable glycemic index; RAG, rapidly available glucose; RDS, rapidly digestible starch; SDS, slowly digestible starch; SHI, starch hydrolysis index.

### 3.2 Image Analysis of Gluten‐Free Cakes

**FIGURE 3:**
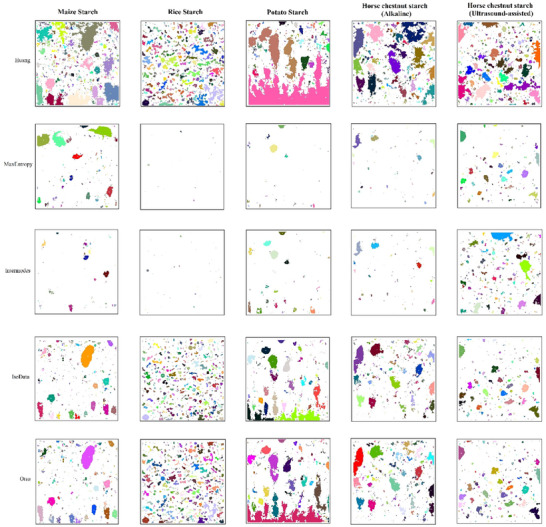
Pore structures produced from gluten‐free cakes using different thresholding methods.

The image processing results of the pore structures of gluten‐free cakes produced with different starch sources are shown in Table 3. Cell count, total area, average cell size, % area, minimum and maximum cell area, cell perimeter, and DFp and DFt values of gluten‐free cakes were determined using Huang, MaxEntropy, Intermodes, Isodata, and Otsu thresholding algorithms. The pore structures of gluten‐free cakes produced with starches differ from each other. The highest cell count was determined by the Huang algorithm, and the lowest cell count was determined by the Intermodes and MaxEntropy algorithms. Gluten‐free cakes made with horse chestnut starch (A‐HCS and U‐HCS) are similar to gluten‐free cakes made with MS when Isodata and Otsu algorithms are considered. When cakes produced using corn starch, rice starch, potato starch, alkaline and ultrasonically produced starches were analyzed, the total area (mm^2^) detected ranged from 267.86 to 330.11, 10.10 to 58.98, 7.14 to 179.66, 100.09 to 192.68, and 111.61 to 232.73 mm^2^ for Huang, MaxEntropy, Intermodes, Isodata, and Otsu algorithms, respectively. A‐HCS was similar to PS and MS, and U‐HCS was similar to rice starch (RS). The average pore size of gluten‐free cakes made with MS ranged from 0.17 to 0.36 mm^2^, whereas it was 0.10–0.41 for RS, 0.40–0.68 for PS, 0.31–0.57 for A‐HCS, and 0.27–0.44 mm^2^ for U‐HCS. PS and A‐HCS cakes and RS and U‐HCS cakes are similar in % area determined from the 3 × 3 cm^2^ image. The Huang algorithm measured the highest % area in all samples, followed by the Otsu, Isodata, MaxEntropy, and Intermodes algorithms (Figure 3). All thresholding algorithms detected pores with a minimum size of 0.002 mm^2^, and smaller values were not detected. Gluten‐free cakes made with PS were found to have larger pores than other cakes. The highest cell perimeter was found in gluten‐free cakes made with potato starch, with an average of 1.92 mm, and the lowest cell perimeter was found in gluten‐free cakes made with MS with an average of 1.37 mm. Figure 3 shows that gluten‐free cakes with PS have a larger pore structure. Studies on the pore structure of cakes and bread are available in the literature. In a study where muffin cakes were made by adding pumpkin flour, the total number of pores in the cakes was reported to be between 264 and 329, and the total cell area was reported to be between 231.32 and 266.26 mm^2^ (Scarton et al. 2021). It was reported that the total number of pores in cakes produced by adding locust (*Locusta migratoria*) and mealworm (*Tenebrio molitor*) powders was 523–617, the average cell size was 0.40–0.53 mm^2^, and the average cell area was 0.42–0.54 mm^2^ (Çabuk 2021). In a study investigating the effect of different enzymes on the technological quality of gluten‐free bread, it was reported that the cell density was between 24 and 35.69 cells/cm^2^ and 42.61% of the pores in the samples were larger than 5 mm (Ebling et al. 2022). The results obtained in our study are similar to those reported in the literature.

**TABLE 3:** Image analysis results of the pore structure of gluten‐free cakes.

		Cell count	Total area (mm^2^)	Average cell size (mm^2^)	% Area	Minimum cell area (mm^2^)	Maximum cell area (mm^2^)	Cell periphery (mm)	DFp	DFt
**Maize starch**	Huang	852.00	303.16	0.36	33.64	0.002	76.86	1.63	1.753	1.304
	MaxEntropy	165.76	58.98	0.17	6.54	0.002	14.95	1.21	1.446	1.056
	Intermodes	75.00	179.66	0.26	19.94	0.002	22.45	1.42	1.532	1.103
	IsoData	362.00	100.09	0.28	11.11	0.002	32.21	1.31	1.750	1.423
	Otsu	407.00	111.61	0.28	12.38	0.002	34.32	1.33	1.721	1.455
**Rice starch**	Huang	685.00	278.55	0.41	30.91	0.002	42.09	2.39	1.151	1.098
	MaxEntropy	133.27	10.10	0.12	1.15	0.002	11.45	1.11	1.183	1.835
	Intermodes	71.00	7.14	0.10	1.00	0.002	11.26	1.05	1.177	1.827
	IsoData	613.00	142.64	0.23	15.83	0.002	17.91	1.70	1.173	1.763
	Otsu	658.00	173.50	0.27	16.25	0.002	19.06	1.84	1.176	1.621
**Potato starch**	Huang	484.00	329.06	0.68	36.51	0.002	213.74	2.08	3.399	0.448
	MaxEntropy	94.16	38.01	0.40	6.88	0.002	32.16	1.75	2.008	0.768
	Intermodes	116.00	47.70	0.43	5.29	0.002	31.37	1.71	2.001	0.761
	IsoData	437.00	192.68	0.46	21.38	0.002	53.96	2.00	1.800	0.881
	Otsu	468.00	232.73	0.52	25.83	0.002	80.46	2.07	2.003	0.765
**Horse chestnut starch (alkaline)**	Huang	582.00	330.11	0.57	36.63	0.002	118.87	2.37	1.839	0.683
	MaxEntropy	73.23	36.45	0.43	3.73	0.002	16.68	1.70	1.996	0.671
	Intermodes	60.00	32.56	0.42	3.61	0.002	16.55	1.69	1.990	0.668
	IsoData	439.00	130.96	0.35	14.98	0.002	25.24	1.47	1.892	1.215
	Otsu	478.00	154.63	0.31	16.94	0.002	25.69	1.51	1.647	1.374
**Horse chestnut starch (ultrasound‐assisted)**	Huang	612.00	267.86	0.44	29.72	0.002	48.79	2.03	1.645	0.996
	MaxEntropy	99.00	29.22	0.27	6.47	0.002	28.64	1.61	1.533	1.121
	Intermodes	113.00	34.86	0.29	4.15	0.002	22.14	1.56	1.513	1.113
	IsoData	358.00	112.10	0.32	12.44	0.002	31.61	1.59	1.593	1.281
	Otsu	392.00	124.64	0.32	13.83	0.002	32.74	1.61	1.592	1.287

Abbreviation: DFp, dimension of each cell.

Products with pores, such as cakes and bread, are so complex and irregular that they cannot be described by classical geometry. A model is needed to describe the irregular structure of such structures. Fractal geometry, used in image analysis and segmentation, is one of the important models developed to characterize the roughness in an image (Chen et al. 2003). In addition, the fractal dimension is related to the human perception of surface roughness. Using the fractal DFp, which is calculated based on the logarithmic relationship between the perimeter (*P*) and area (*A*) of the cells, and the image entropy and texture fractal dimension selected for fractal evaluation, the DFt values used in the cell counting method were calculated from grayscale images and are shown in Table 3. The highest fractal dimension was found in PS, and the lowest in RS. The higher the fractal dimension, the rougher the measured area (Chen et al. 2003). A‐HCS and U‐HCS are similar to MS according to the fractal dimensions of the pore structures. Gluten‐free cakes made with horse chestnut starch were similar to rice and corn starch in terms of pore structure. It was found that Huang, Isodata, and Otsu thresholding algorithms were more effective in evaluating the pore structure of gluten‐free cakes with a high proportion of small pores (<5 mm), whereas MaxEntropy and Intermodes algorithms were insufficient.

## 4 Conclusion

This study deals with the production of starch, a high value‐added component, from horse chestnut, a waste material, and the production of gluten‐free cake from horse chestnut starch. Two different methods, alkaline and ultrasound‐assisted, were used for starch production. The physical, chemical, nutritional, and textural properties of gluten‐free cakes made from horse chestnut starch and gluten‐free cakes made from rice, maize, and potato starches were determined. In addition, the pore structure, which is one of the most important quality criteria of gluten‐free cakes, was determined by image processing methods using five different thresholding algorithms. Gluten‐free cakes made with horse chestnut starch were similar to rice starch in terms of physical and textural properties, to MS in terms of slowly digestible starch and PGI, and to rice starch in terms of pore structure. The Huang, Isodata, and Otsu thresholding algorithms were found to be more efficient in assessing the pore structure of gluten‐free cakes with a high proportion of small pores (<5 mm), whereas the MaxEntropy and Intermodes algorithms were found to be inadequate. This study is a comprehensive comparative study of a new starch source for gluten‐free cake production. It is believed that the information obtained from this study can guide researchers. Use in the production of other gluten‐free products such as biscuits and bread and consumer preference research are recommended.

## Author Contributions

**Ali Cingöz**: methodology, writing–review and editing, writing–original draft, project administration, conceptualization, visualization.

## Conflicts of Interest

The author declares no conflicts of interest.
